# Effects of Inpatient Occupational Rehabilitation vs. Outpatient Acceptance and Commitment Therapy on Sick Leave and Cost of Lost Production: 7-Year Follow-Up of a Randomized Controlled Trial

**DOI:** 10.1007/s10926-024-10195-x

**Published:** 2024-04-28

**Authors:** Lene Aasdahl, Sigmund Østgård Gismervik, Roar Johnsen, Ottar Vasseljen, Gudrun M. W. Bjørnelv, Johan Håkon Bjørngaard, Marius Steiro Fimland

**Affiliations:** 1 Department of Public Health and Nursing, Faculty of Medicine and Health Sciences, Norwegian University of Science and Technology, Postboks 8905, 7491 Trondheim, Norway; 2 Unicare Helsefort Rehabilitation Centre, Rissa, Norway; 3 Clinic of Rehabilitation, St. Olavs Hospital, Trondheim University Hospital, Trondheim, Norway; 4 Department of Health Management and Health Economics, Faculty of Medicine, University of Oslo, Oslo, Norway; 5 Faculty of Nursing and Health Sciences, Nord University, Levanger, Norway; 6 Department of Neuromedicine and Movement Science, Faculty of Medicine and Health Sciences, Norwegian University of Science and Technology, Trondheim, Norway

**Keywords:** Return-to-work, Sick leave, Musculoskeletal diseases, Mental health, Cognitive therapy

## Abstract

**Objectives:**

Previously, we reported that an inpatient multimodal occupational rehabilitation program (I-MORE) was more effective than outpatient Acceptance and Commitment Therapy (O-ACT) in reducing sickness absence and was cost-effective over a 24-month period. Here we present 7-years of follow-up on sick leave and the cost of lost production.

**Methods:**

We randomized individuals aged 18–60, sick-listed due to musculoskeletal or mental health disorders to I-MORE (*n* = 82) or O-ACT (*n* = 79). I-MORE, lasting 3.5 weeks, integrated ACT, physical training, and work-related problem-solving. In contrast, O-ACT mainly offered six weekly 2.5 h group sessions of ACT. We measured outcomes using registry data for days on medical benefits and calculated costs of lost production. Our analysis included regression analyses to examine differences in sickness absence days, logistic general estimating equations for repeated events, and generalized linear models to assess differences in costs of lost production.

**Results:**

Unadjusted regression analyses showed 80 fewer days of sickness absence in the 7-year follow-up for I-MORE compared to O-ACT (95% CI − 264 to 104), with an adjusted difference of 114 fewer days (95% CI − 298 to 71). The difference in costs of production loss in favour of I-MORE was 27,048 euros per participant (95% CI − 35,009 to 89,104).

**Conclusions:**

I-MORE outperformed O-ACT in reducing sickness absence and production loss costs during seven years of follow-up, but due to a limited sample size the results were unprecise. Considering the potential for substantial societal cost savings from reduced sick leave, there is a need for larger, long-term studies to evaluate return-to-work interventions.

**Supplementary Information:**

The online version contains supplementary material available at 10.1007/s10926-024-10195-x.

## Introduction

Sick leave can severely affect an individual’s quality of life, social role, and financial situation [1, 2]. In addition, sick leave has vast impacts on societal productivity [2], and is expected to increase in the future due to demographic changes with an aging population. There has been considerable research on interventions to facilitate return-to-work (RTW), with some reports of positive long-term effects on sick leave and costs [3–5]. However, outcomes vary, and there are few studies with long-term follow-up [4, 6].

We have previously reported two-year follow-up data comparing a 3.5-week inpatient multimodal occupational rehabilitation (I-MORE) with a less comprehensive outpatient Acceptance and Commitment Therapy (O-ACT) program for individuals sick-listed due to musculoskeletal or mental health disorders [7–9]. We observed fewer days of sickness absence for I-MORE compared to O-ACT [7, 8]. We also found that I-MORE, despite considerably higher interventions costs, was cost-effective compared to O-ACT in a societal perspective due to lower production loss [9]. This paper provides an updated analysis with 7-years follow-up on sick leave and costs of lost production.

## Methods

### Study Design, Participants, and Interventions

We conducted a randomized controlled trial with parallel groups comparing the effect of I-MORE to O-ACT on sickness absence and production loss over seven years. The primary outcome was sickness absence during 12 months of follow-up, and is reported previously [7].

Eligible participants were 18 to 60 years of age and sick-listed 2 to 12 months with a diagnosis within the musculoskeletal (L), psychological (P) or general and unspecified (A) categories of the ICPC-2 (International Classification of Primary Care, Second edition). I-MORE consisted of physical training, mindfulness, psychoeducation, ACT [10], and work-related problem-solving, conducted both individually and in groups. This program lasted 3.5 weeks, involving 6–7 h daily except on weekends. O-ACT primarily featured group-based ACT-sessions, lasting 2.5 h weekly for six weeks. In addition, there was a group session with psychoeducation on physical activity, two individual sessions with a social worker, and a short individual closing session with a group therapist. Further details about the trial and the interventions have been reported previously [7, 11].

### Outcome Measures

Sick leave data were obtained from the Norwegian National Social Security System Registry, where all individuals receiving any form of sickness or disability benefits in Norway are registered by their social security number. To calculate days of sick leave we included the different types of medical benefits: sick leave payments, work assessment allowance and disability pension. Production loss, reflecting the 7-year period, was calculated by multiplying the number of sickness absence days with the average daily wage of 339 euros, as of 2016 data from Statistics Norway [12].

### Other Variables

Descriptive variables registered by questionnaires at inclusion were anxiety and depression symptoms, measured using The Hospital Anxiety and Depression scale (HADS) [13], pain assessed by one question from the Brief Pain Inventory (BPI) [14], and educational attainment, categorized as high (college/university) or low. Information about age and sex was obtained from registry data.

### Randomization and Blinding

Potential participants were identified in the National Social Security System and randomized after an outpatient screening. An electronic randomization procedure was provided by the Unit of Applied Clinical Research (third-party) at the Norwegian University of Science and Technology (NTNU). Blinding of participants and caregivers was not possible. Sickness absence data were provided by the Norwegian Welfare and Labour Service, who was unaware of group allocation. The researchers were not blinded.

### Statistical Analysis

Sample size was calculated based on the primary outcome, i.e., number of sickness absence days during 12 months of follow-up resulting in 80 persons in each arm [11]. In the current study, to account for the actual follow-up durations, i.e., time until retirement, death, or 7 years of follow-up, we standardized sickness absence days for each participant. This was done by first calculating a ratio of number of sickness absence days over possible sickness absence days for seven years for each participant. The ratio was then multiplied by the possible workdays during follow-up (260 days per year). Calculations were based on a 5-day work week and accounted for part-time positions. All three types of medical benefits (sick leave payments, work assessment allowance and disability pension) were used in the calculations. If a participant received a graded disability at inclusion this was not considered sick leave (as it is a permanent benefit), but any subsequent increase in disability benefits were included.

We used regression analyses to estimate the difference in sickness absence days between the groups. The analyses were performed unadjusted and adjusted for age (continuous), sex, education level (high/low) and diagnosis (musculoskeletal/mental health including general and unspecified) for sick leave. The probability of not receiving any medical benefits each month (i.e., working) during follow-up was analyzed as repeating events with logistic general estimating equations. We used an exchangeable correlation structure and robust standard errors. Between group differences in total costs of lost production was tested using generalized linear models with a log link and a gamma distribution. The analyses were performed unadjusted and adjusted (with the beforementioned variables). All analyses were performed in line with the intention-to-treat principle. Precision was assessed using 95% confidence intervals. STATA 17 was used for all analyses (StataCorp. 2021. Stata Statistical Software: Release 17. College Station, TX: StataCorp LP).

## Results

In total 166 participants were randomized to I-MORE (*n* = 86) and O-ACT (*n* = 80). Five participants (four in I-MORE, one in O-ACT) declined the 7-year follow-up, leaving 161 participants (82 and 79). The baseline characteristics for participants in the two groups were similar (Table 1).

**Table 1:** Baseline characteristics for participants

	I-MORE (*n* = 82)	O-ACT (*n* = 79)
Age mean (SD)	46.5 (8.6)	45.2 (10.4)
Women *n* (%)	66 (81%)	60 (76%)
Higher education^a^ *n* (%)	30 (37%)	34 (44%)
Work status *n* (%)		
No work	11 (13)	6 (8)
Full time	51 (62)	53 (67)
Part time	11 (13)	17 (22)
Graded disability pension	9 (11)	3 (4)
Sick leave status^b^ *n* (%)		
Full sick leave	32 (39%)	37 (47%)
Partial sick leave	44 (54%)	36 (46%)
Work assessment allowance	6 (7%)	6 (8%)
Main diagnoses for sick leave (ICPC-2)^b^ *n* (%)		
L-musculoskeletal	53 (65%)	40 (51%)
P-psychological/A-general and unspecified	29 (36%)	39 (49%)
Length of sick leave at inclusion^b,c^		
Median days (IQR)	204 (163–265)	215 (176–262)
Pain level, mean (SD)	5.0 (2.1)	4.8 (2.1)
HADS mean (SD)^d^		
Anxiety (0–21)	7.3 (3.9)	8.6 (4.1)
Depression (0–21)	5.6 (4.0)	6.6 (4.0)

*I-MORE* inpatient multimodal occupational rehabilitation, *O-ACT* outpatient acceptance and commitment therapy

^a^Higher education: college or university

^b^From National Social Security System Registry

^c^Sick leave days in past 12 months prior to inclusion, measured as calendar days, not adjusted for graded sick leave or part-time job

^d^Measured by the Hospital Anxiety and Depression Scale

Over the 7-year follow-up, the median total number of sickness absence days were 689 for I-MORE (IQR 254 to 1156) and 770 for O-ACT (IQR 287 to 1422). The mean number of sickness absence days were 761 (SD 570) and 840 (SD 611), respectively. The unadjusted regression analyses showed that I-MORE resulted in 80 fewer sickness absence days (95% CI − 264 to 104) for I-MORE compared to O-ACT. The adjusted analyses showed 114 fewer days (95% CI − 298 to 71) for I-MORE.

**Fig. 1:**
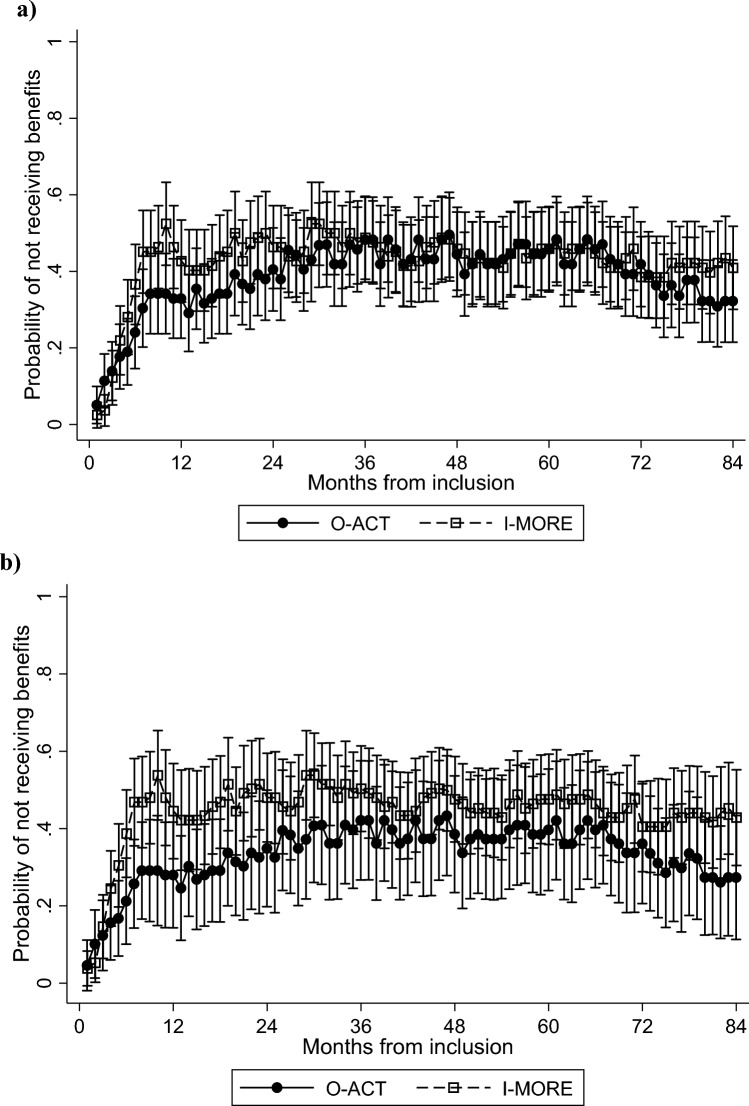
Monthly estimated probability of no medical benefits for inpatient multimodal occupational rehabilitation (I-MORE) versus outpatient acceptance and commitment therapy (O-ACT), based on logistic general estimating equations analyses. **a** unadjusted; **b** adjusted for age, sex, education, and main sick leave diagnosis

Monthly unadjusted estimates showed that I-MORE participants were more likely to be without medical benefits (i.e., working) in the first year, levelling off over time (Fig. 1a). In adjusted analyses this convergence began around the third year (Fig. 1b).

Over the 7-year period, 79% of the participants in I-MORE and 68% in O-ACT achieved sustainable RTW, i.e., one month without medical benefits. Many participants transitioned between work and different types of benefits during follow-up (Online Supplementary Table S1). At the 7-year mark, 40% were working in the I-MORE group and 29% in the O-ACT group. Full permanent disability benefits were received by 21% in the I-MORE group and 28% in O-ACT. During follow-up, five participants were retired (I-MORE *n* = 1; O-ACT *n* = 4) and four died (2 in each group).

Over seven years, the mean costs of lost production for I-MORE were 257,845 euros (SD 193,287) compared to 284,893 euros (SD 207,044) for O-ACT. This difference favoured I-MORE by 27,048 euros per participant (95% CI − 35,009 to 89,104) in unadjusted analyses, and 46,891 euros (95% CI − 19,190 to 112,972) in adjusted analyses. Cumulative costs over the seven years of follow-up are graphically presented in Online Supplementary Figure S1.

## Discussion

I-MORE outperformed O-ACT in reducing sickness absence days over seven years with 80–114 days, and consequently incurred lower costs of lost production. However, these results should be interpreted with caution due to the limited sample size.

While few studies have assessed long-term RTW intervention outcomes or incorporated economic evaluations [4, 6], some research aligns with our findings. The impact of I-MORE on sickness absence was most pronounced in the initial years after rehabilitation, consistent with prior research showing the most pronounced effects to occur within three years after rehabilitation [3, 15]. Our participants had on average 200 days of sickness absence at study inclusion which underscores the likely complexity of health issues and challenges with work participation in this study population.

The multicomponent nature of I-MORE precludes identifying which elements contribute to its effectiveness. Key distinctions between the programs include I-MORE`s inpatient format, greater intensity, and multimodal approach. Moreover, although the 3.5-week I-MORE intervention might be perceived as expensive, especially by policymakers who favor less resource demanding programs, it is important to consider the overall value it provides. An attempt to create a condensed, less costly 8-day version of I-MORE, revealed that it did not outperform O-ACT in terms of RTW [16]. In contrast, the 3.5-week I-MORE program, despite its higher cost, was cost-effective in a societal perspective compared to O-ACT [9]. A challenge when implementing RTW programs in Norway is that the costs fall on the healthcare system while the savings (reduced sick leave) benefit another sector. The results of this study, and previous publications from this project, highlights the need for policymakers to adopt a societal perspective when planning interventions for sick-listed workers to consider broader economic benefits. This also aligns with recommendations by an expert group on priority setting in Norway [17].

The vast societal impact of sick leave means that even moderate effects of interventions may reduce societal costs substantially. Therefore, the low precision in our effect estimates emphasize the need for larger studies, which could also identify subgroups best suited for comprehensive programs or alternative interventions.

The main strength of this study was the use of long-term registry data for sickness absence, eliminating missing data and recall bias. The main limitation was the limited sample size.

In summary, while I-MORE outperformed O-ACT in reducing sickness absence days and also costs of lost production, the low precision of the estimates, due to a small sample size, remains a concern. Given the societal implications of extended sick leave, future larger-scale RTW intervention studies with long-term follow-up are encouraged for more accurate effect estimations.

## Supplementary Information

Below is the link to the electronic supplementary material.

Supplementary file1 (PDF 201 KB)

## Data Availability

Data are not available due to ethical approval.
