# Supplementary material for: Effects of Inpatient Occupational Rehabilitation vs. Outpatient Acceptance and Commitment Therapy on Sick Leave and Cost of Lost Production: 7-Year Follow-Up of a Randomized Controlled Trial
Source: J Occup Rehabil. 2024 Apr 28;35(2):288–93. doi: 10.1007/s10926-024-10195-x (PMC12089147; doi:10.1007/s10926-024-10195-x)
Supplement: Supplementary file 1 — Supplementary file1 (PDF 201 KB) [file 10926_2024_10195_MOESM1_ESM.pdf]

Online supplementary material

Table S1: Annual status update on medical benefits, retirement, and deaths, presented as percentages (and counts, n).

	**Status**						
	**No medical benefits (working)**	**Sick listed**	**Work assessment allowance**	**Graded permanent disability benefits (<100%)[a]**	**Full permanent disability benefits (100%)**	**Retired**	**Dead**
**Rehabilitation start[b]**							
I-MORE	1 (1)	91 (75)	7 (6)				
O-ACT		92 (73)	8 (6)				
**Year 1**							
I-MORE	43 (35)	13 (11)	44 (36)				
O-ACT	33 (26)	4 (3)	63 (50)				
**Year 2**							
I-MORE	46 (38)	13 (11)	35 (29)	2 (2)	2 (2)		
O-ACT	41 (32)	6 (5)	46 (36)	4 (3)	4 (3)		
**Year 3**							
I-MORE	49 (40)	17 (14)	21 (17)	9 (7)	5 (4)		
O-ACT	48 (38)	5 (4)	23 (18)	13 (10)	10 (8)		1 (1)
**Year 4**							
I-MORE	45 (37)	13 (11)	20 (16)	10 (8)	11 (9)		1 (1)
O-ACT	43 (34)	11 (9)	16 (13)	13 (10)	14 (11)		3 (2)
**Year 5**							
I-MORE	45 (37)	12 (10)	7 (6)	15 (12)	20 (16)		1 (1)
O-ACT	44 (35)	10 (8)	8 (6)	11 (9)	24 (19)		3 (2)
**Year 6**							
I-MORE	38 (31)	16 (13)	10 (8)	16 (13)	20 (16)		1 (1)
O-ACT	41 (32)	14 (11)	4 (3)	11 (9)	28 (22)		3 (2)
**Year 7**							
I-MORE	40 (33)	13 (11)	6 (5)	16 (13)	21 (17)	1 (1)	2 (2)
O-ACT	29 (23)	14 (11)	9 (7)	13 (10)	28 (22)	5 (4)	3 (2)

Abbreviations: I-MORE: inpatient multimodal occupational rehabilitation; O-ACT: outpatient acceptance and commitment therapy

[a] Only new disability benefits during follow-up are considered. At inclusion, 12 participants received graded disability benefits (I-MORE n=9; O-ACT n=3); they are registered as part-time workers with corresponding benefits.

b) Benefits status the month the participant started rehabilitation. There are some small differences from table 1 which shows status at inclusion in the study.

a)

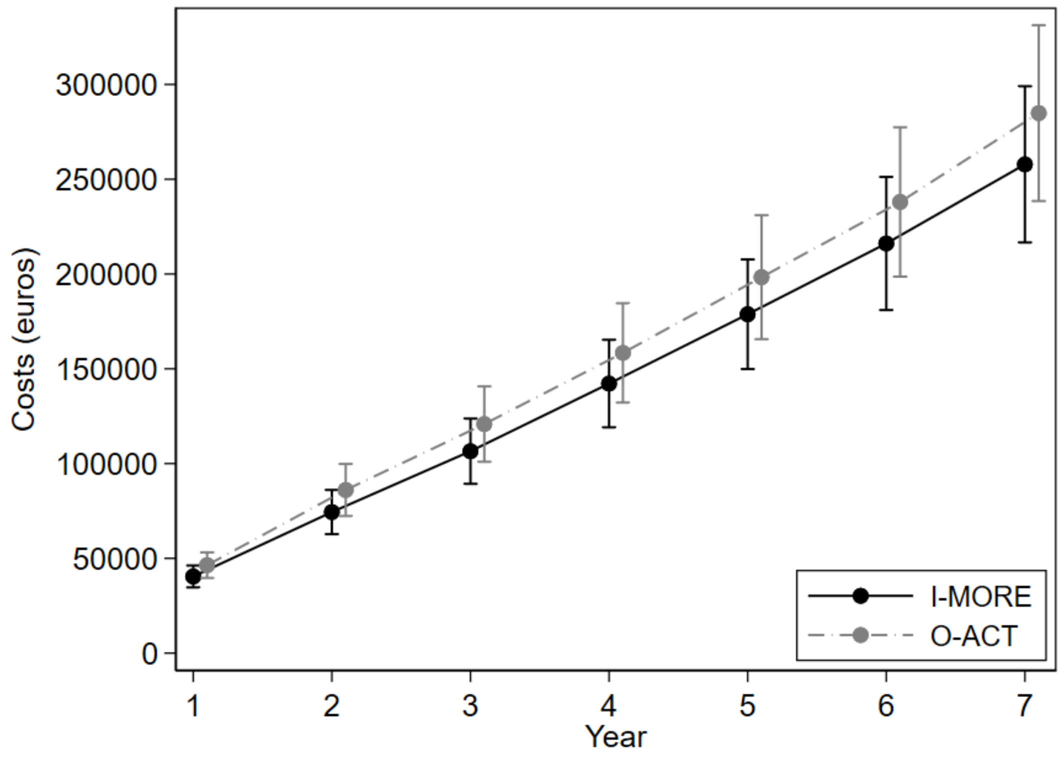

b)

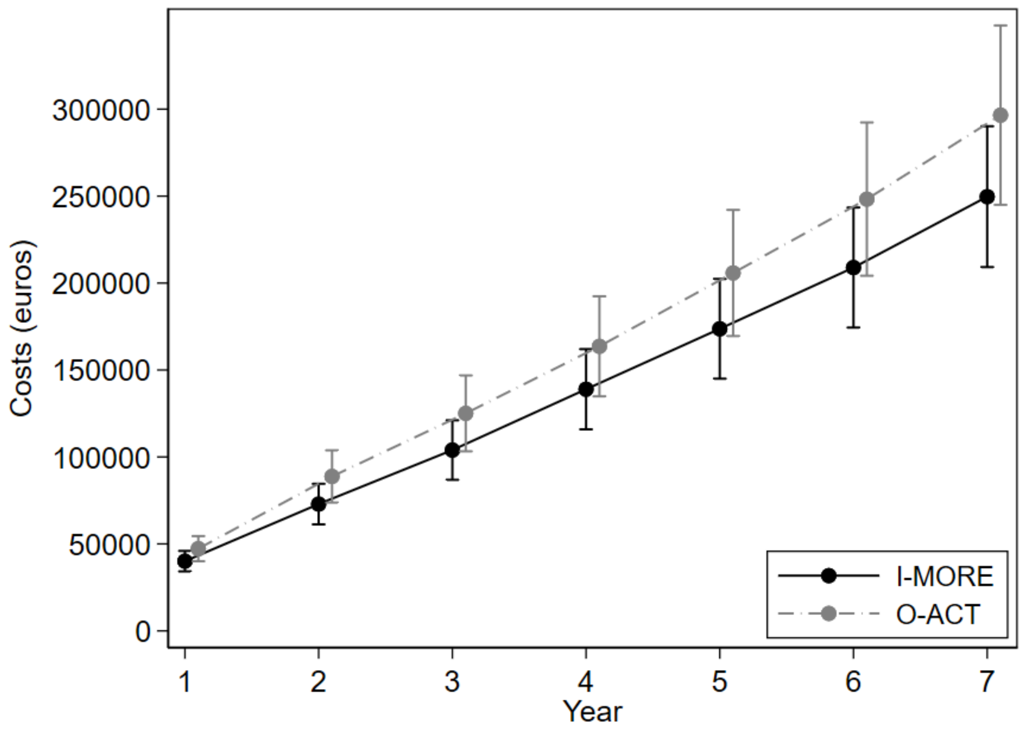

Figure S1 Cumulative costs (in euros) for the inpatient (I-MORE)- and the outpatient program (O-ACT) during 7 years of follow-up. a) unadjusted; b) adjusted for age, sex, education, and main sick leave diagnosis.
